# Supplementary material for: Structural and Thermal Analysis of Softwood Lignins from a Pressurized Hot Water Extraction Biorefinery Process and Modified Derivatives
Source: Molecules. 2019 Jan 18;24(2):335. doi: 10.3390/molecules24020335 (PMC6359013; doi:10.3390/molecules24020335)
Supplement: Supplementary file 1 [file molecules-24-00335-s001.pdf]

Thermal and Structural Analysis of Softwood Lignin from a Pressurized Hot Water Extraction Biorefinery Process and Their Modified Derivatives

Supporting data

Content:

Figure S1. HSQC of acetylated birch lignin
Figure S2. HSQC of acetylated pine lignin
Figure S3. HSQC of acetylated spruce lignin
Figure S4. HSQC of partially methylated birch lignin
Figure S5. HSQC of partially methylated pine lignin
Figure S6. HSQC of partially methylated spruce lignin
Figure S7. HSQC of fully methylated pine lignin
Figure S8. ^{31}P NMR of the BLN softwood lignin and MWL
Figure S9. ^{31}P NMR of the partially methylated BLN lignin
Figure S10. ^{31}P NMR of the fully methylated BLN pine lignin
Figure S11. ^{31}P NMR of the acetylated BLN lignin
Figure S12. ^{31}P NMR of the softwood lignin fractions besides the BLN lignin (MTBE insoluble)
Figure S13. ^{13}C NMR of pine BLN and MWL
Figure S14. ^{13}C NMR of spruce BLN and MWL
Figure S15. ^{13}C NMR of acetylated spruce and pine BLN lignin
Figure S16. TGA of unmodified pine lignin and acetylated pine lignin

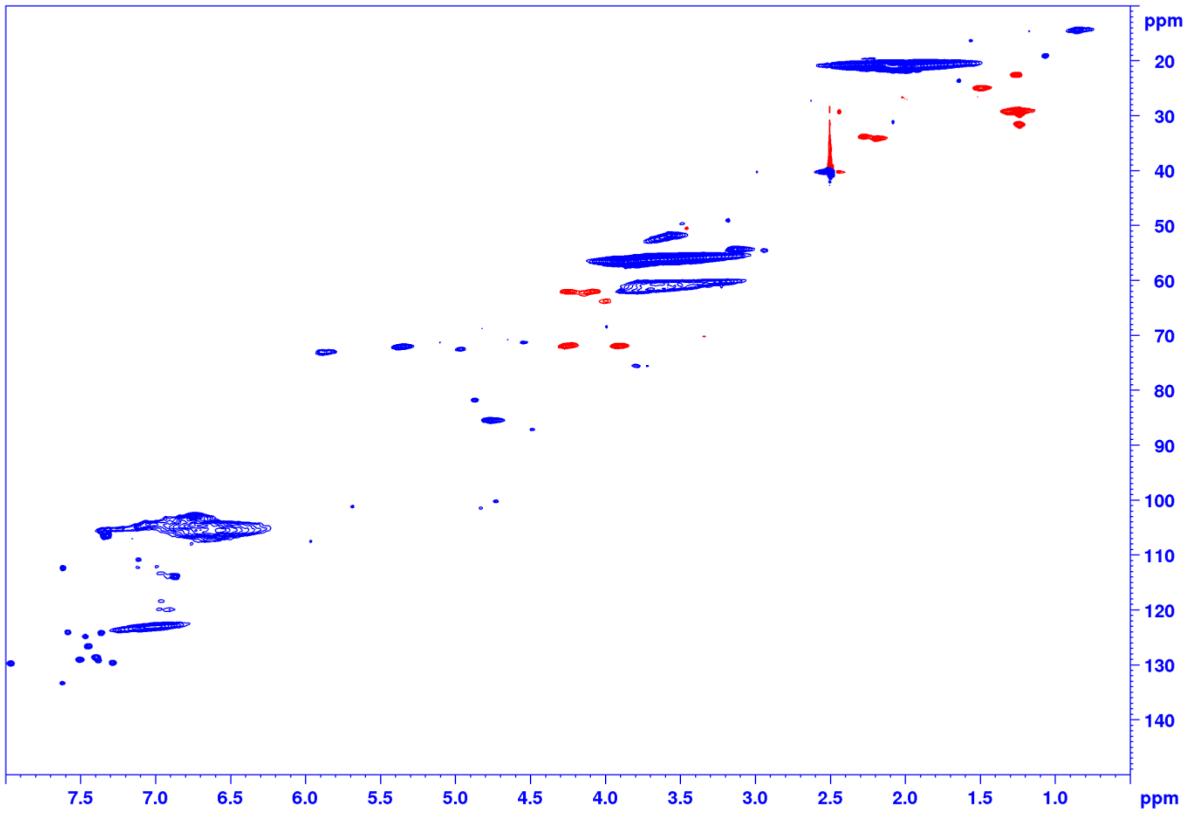

Figure S1. Multiplicity edited HSQC of acetylated birch lignin

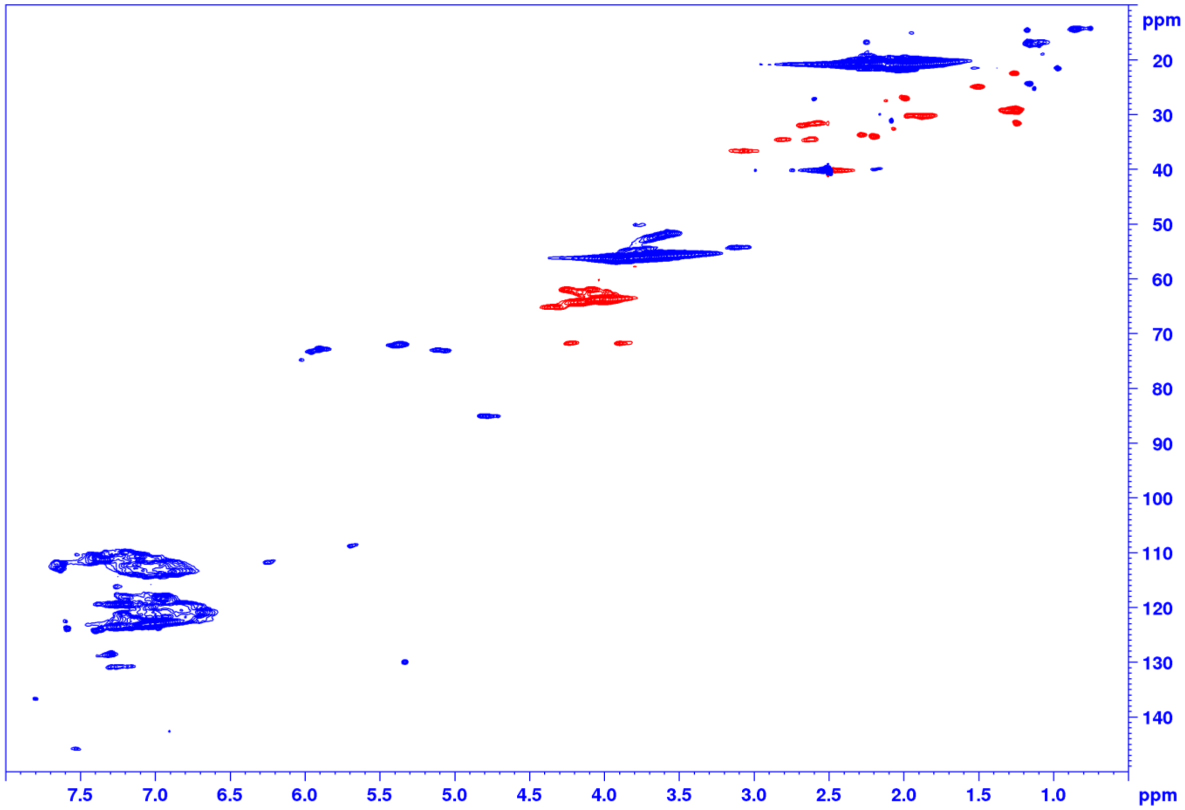

Figure S2. Multiplicity edited HSQC of acetylated pine lignin

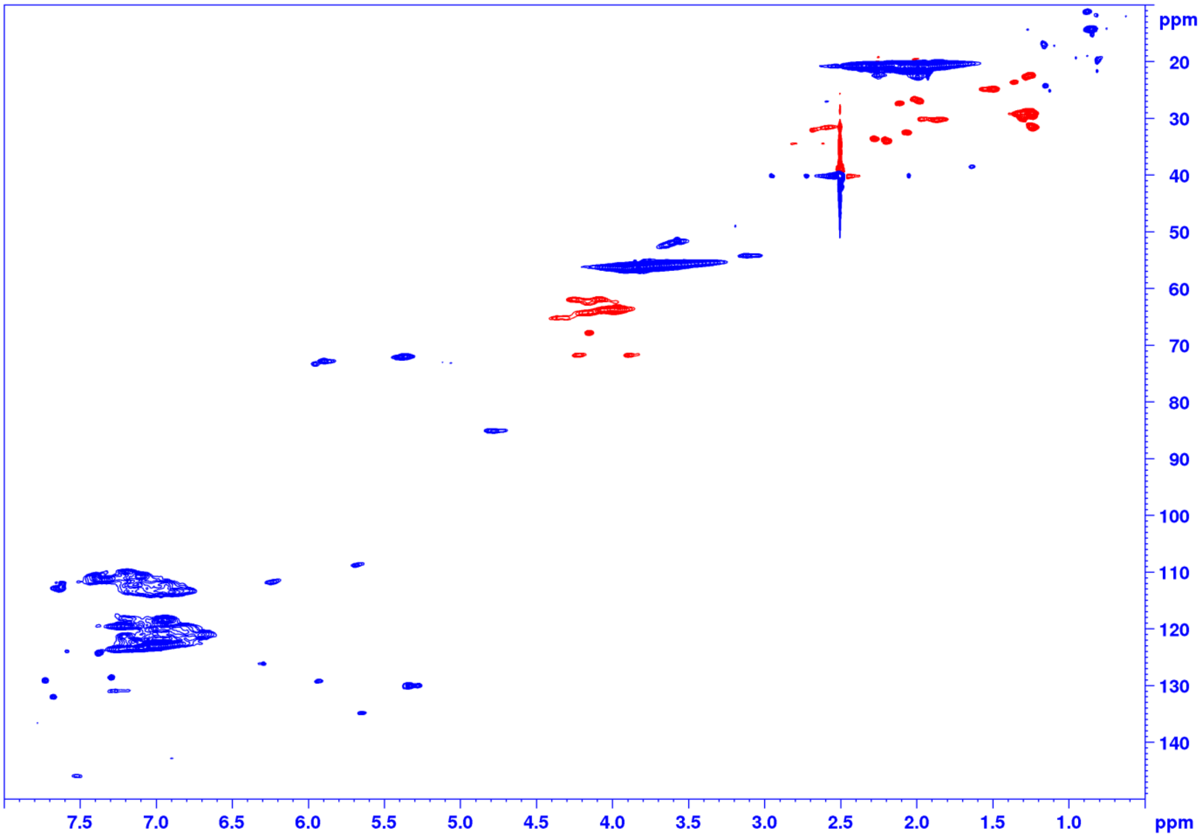

Figure S3. Multiplicity edited HSQC of acetylated spruce lignin

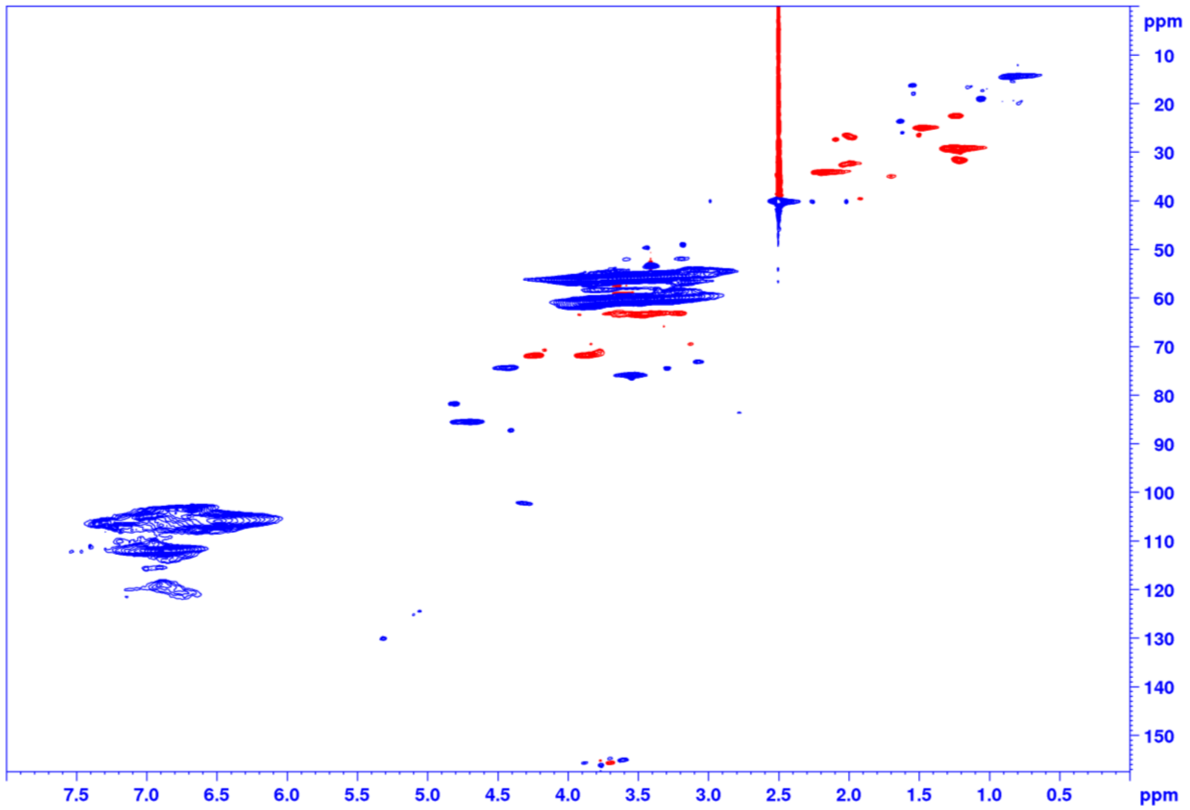

Figure S4. Multiplicity edited HSQC of partially methylated birch lignin

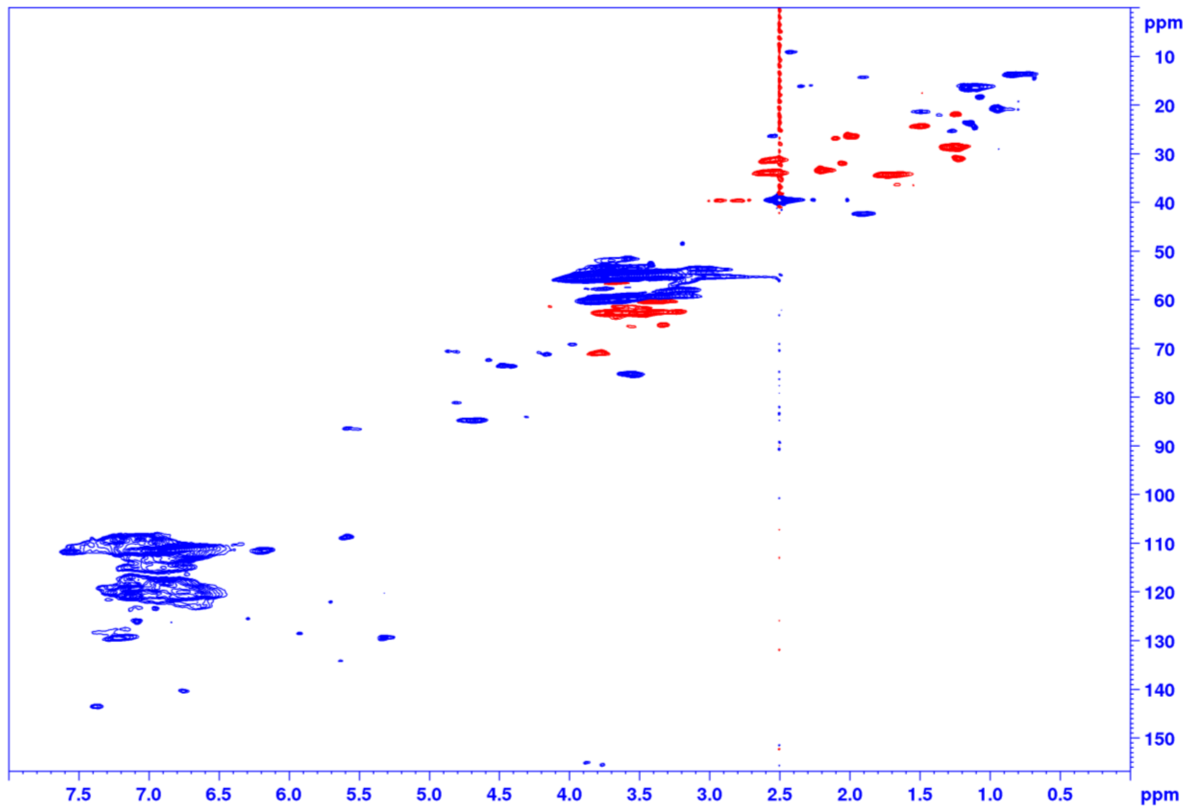

Figure S5. Multiplicity edited HSQC of partially methylated pine lignin

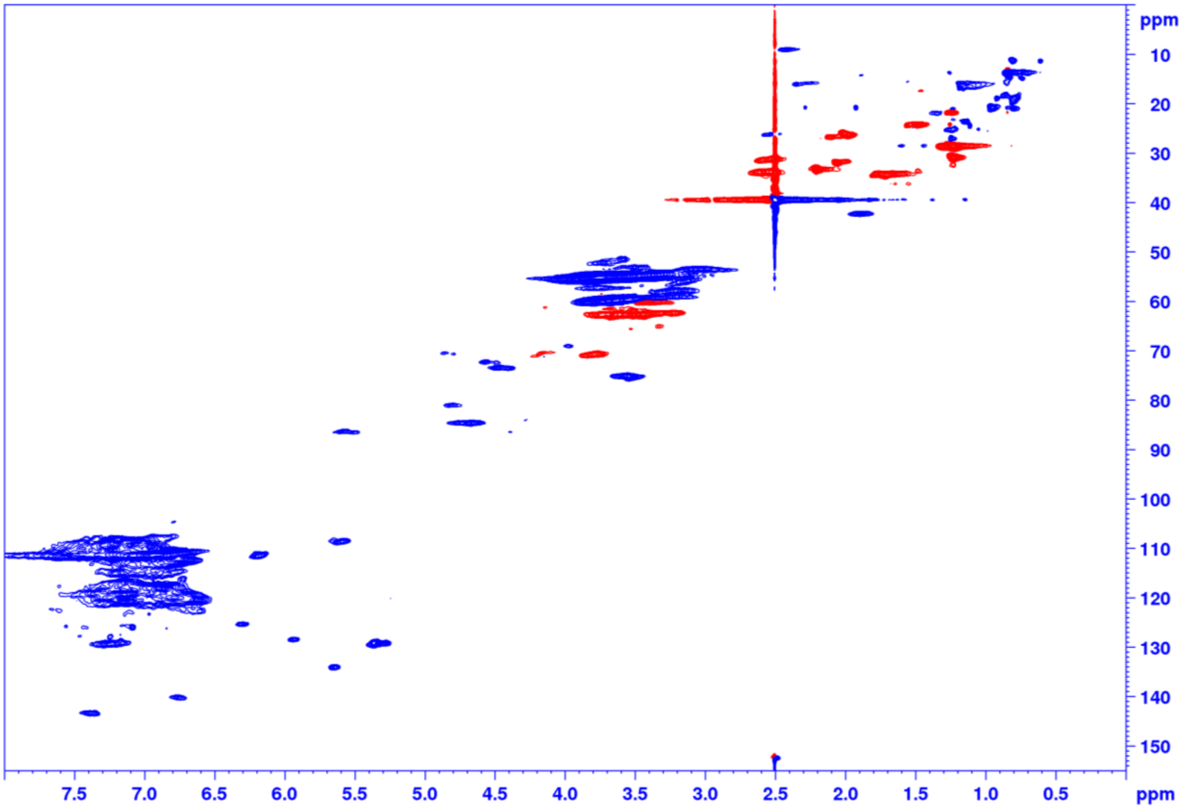

Figure S6. Multiplicity edited HSQC of partially methylated spruce lignin

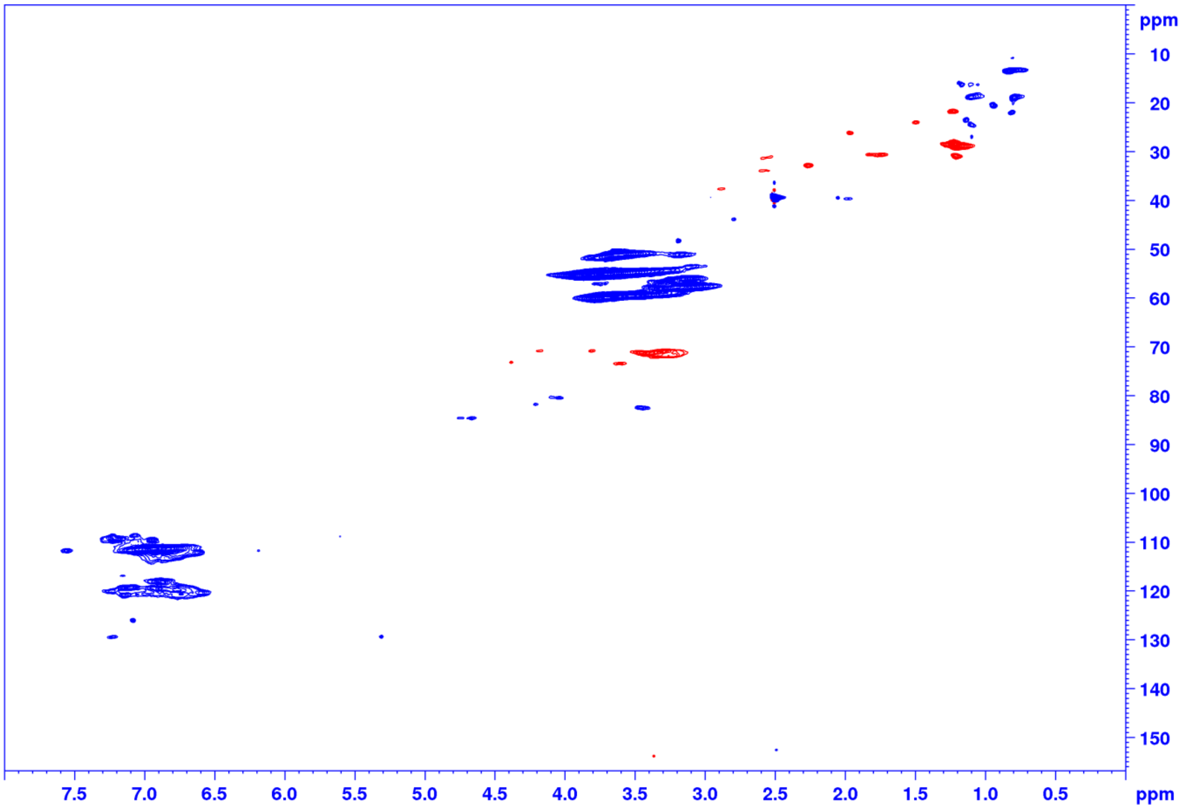

Figure S7. Multiplicity edited HSQC of fully methylated pine lignin

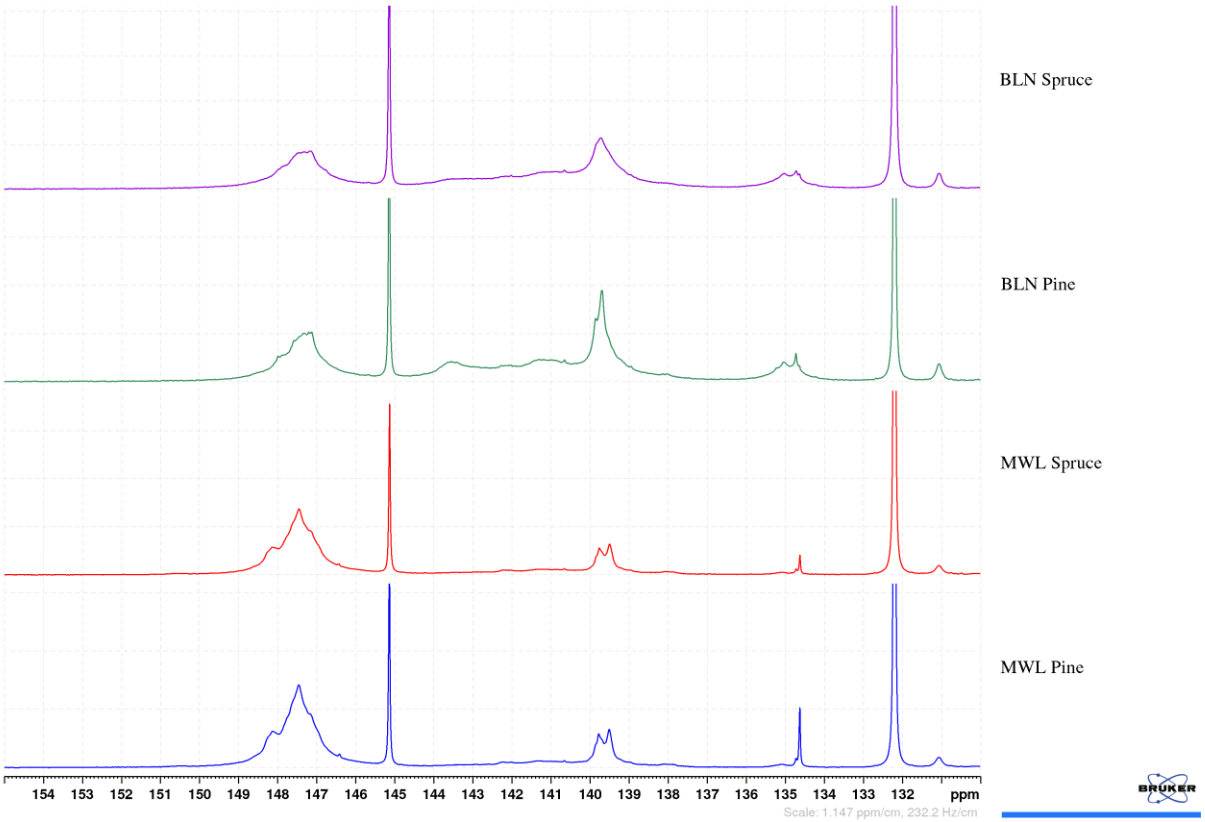

Figure S8. ^{31}P NMR of BLN spruce, BLN pine, MWL spruce and MWL pine

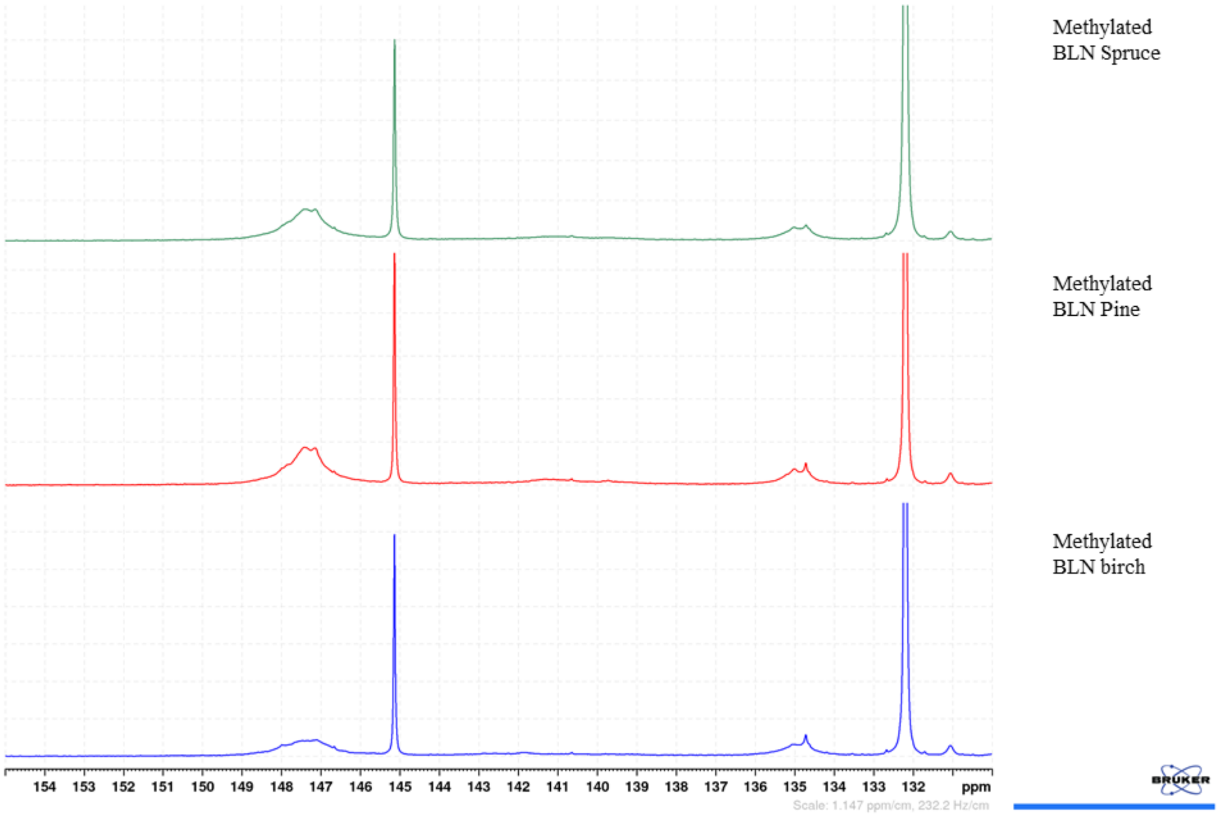

Figure S9. ^{31}P NMR of the lignin with methylated phenolic hydroxyl groups birch, spruce and pine

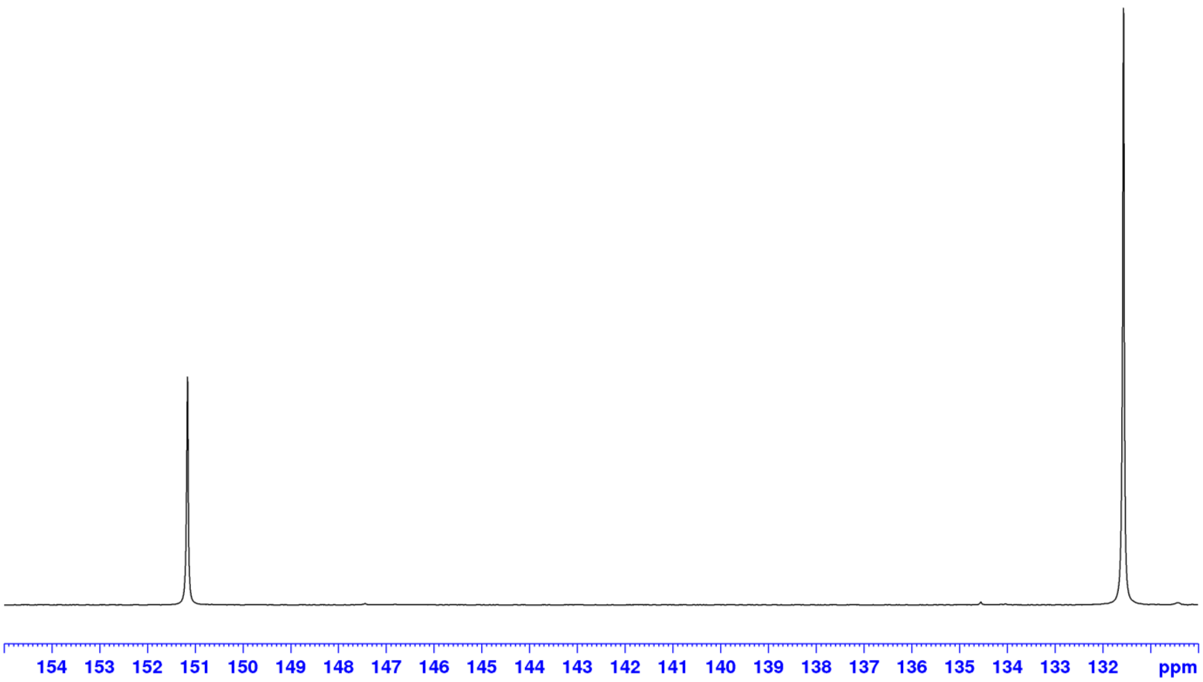

Figure S10. ^{31}P NMR of pine lignin fully methylated

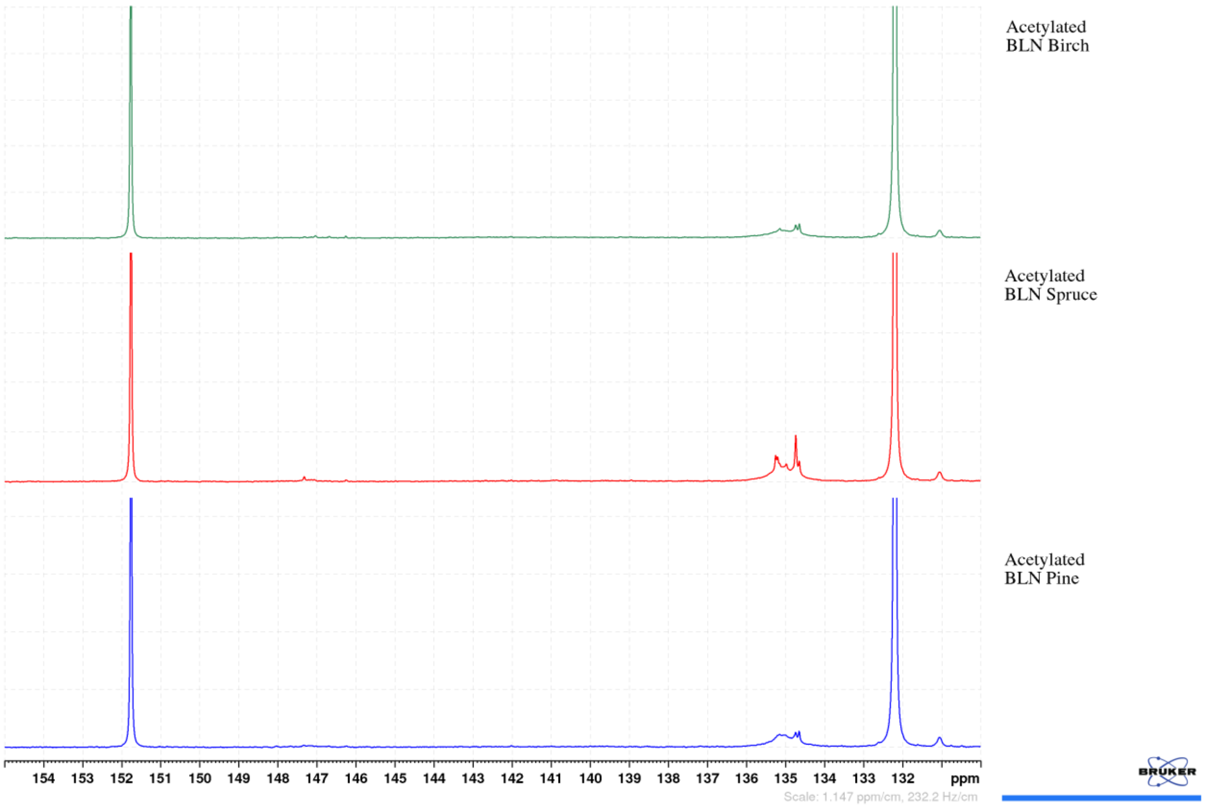

Figure S11. ^{31}P NMR of the acetylated lignin birch, spruce and pine

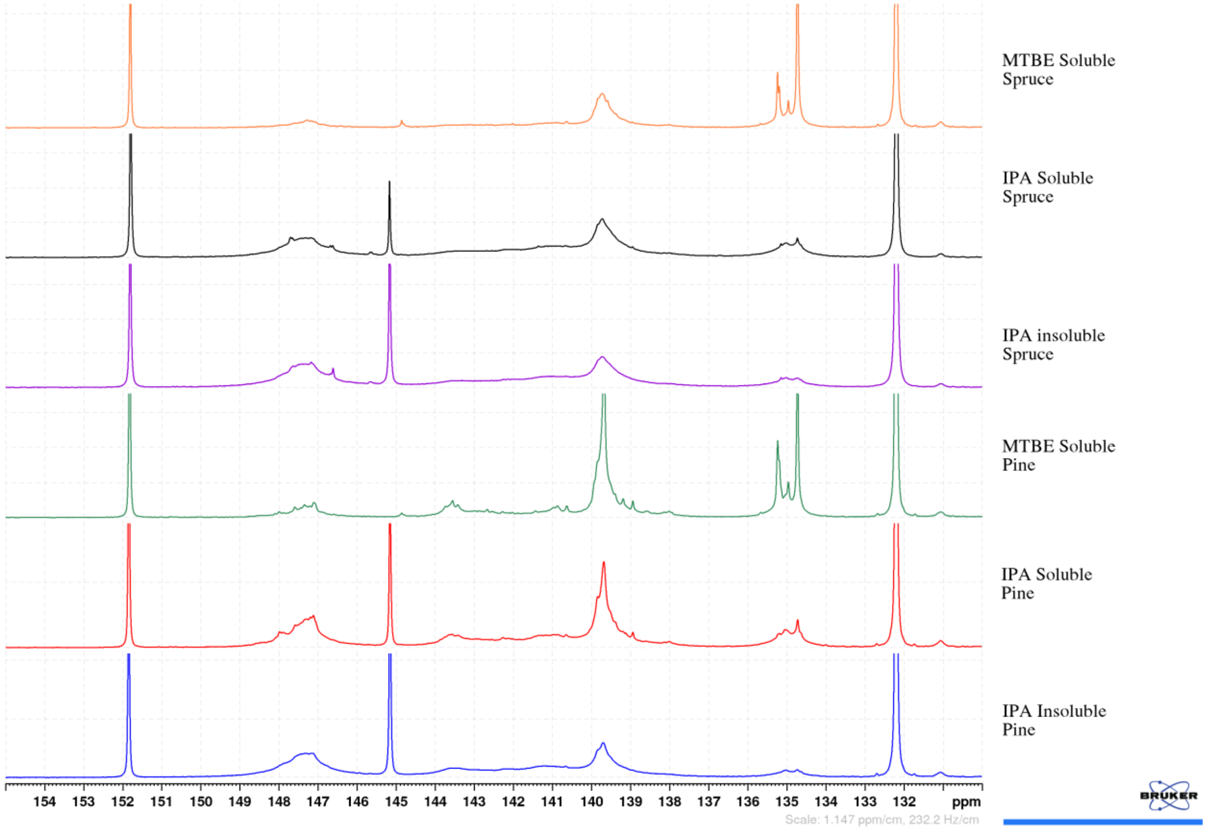

Figure S12. ^{31}P NMR of the different lignin fractions: birch, MTBE soluble spruce, IPA soluble spruce, IPA insoluble spruce, MTBE soluble pine, IPA soluble pine and IPA insoluble pine

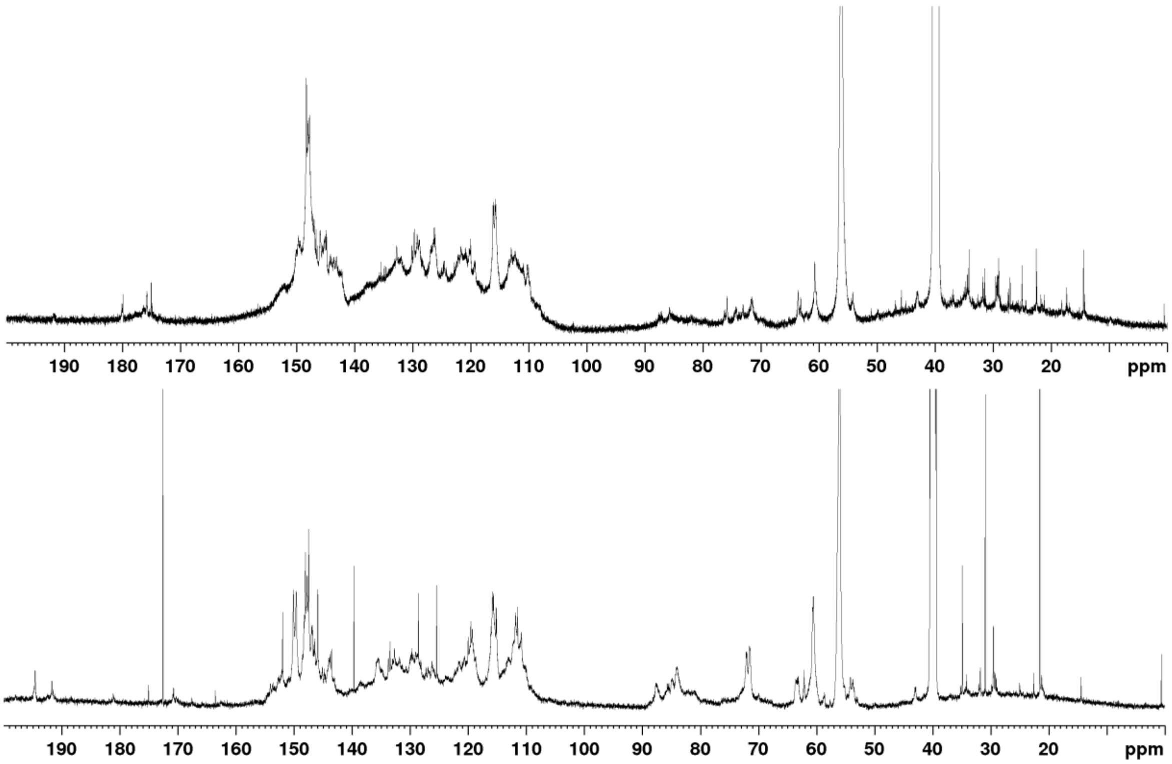

Figure S13. ^{13}C NMR of pine, BLN (top), MWL (down)

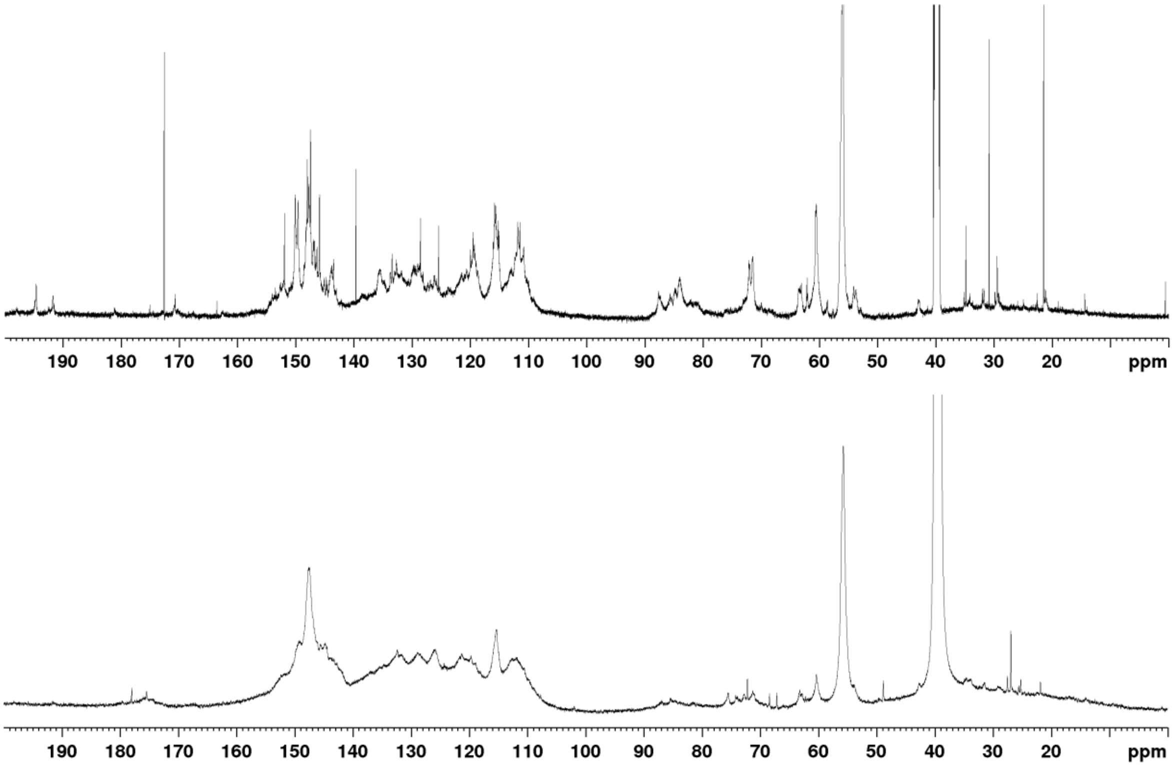

Figure S14. ^{13}C NMR of spruce, BLN (top), MWL (down)

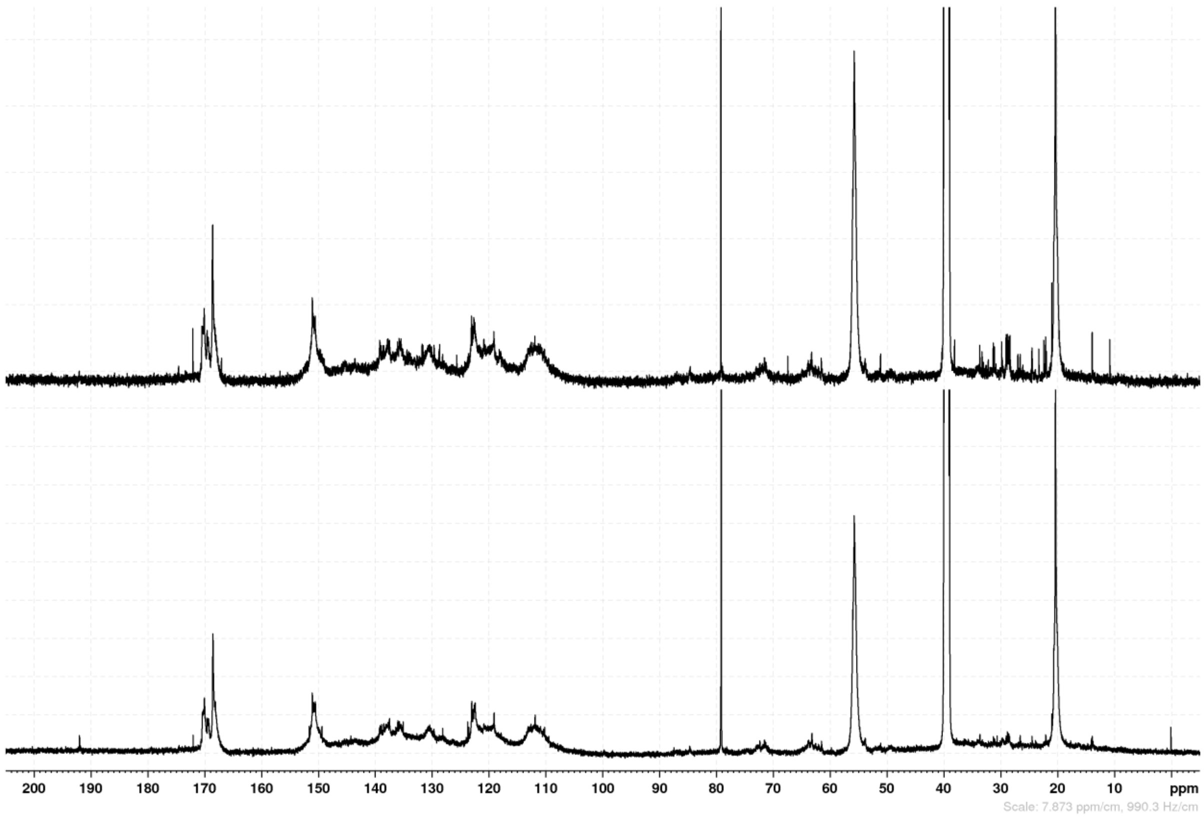

Figure S15. ^{13}C NMR of acetylated BLN lignin spruce (top) and pine (down)

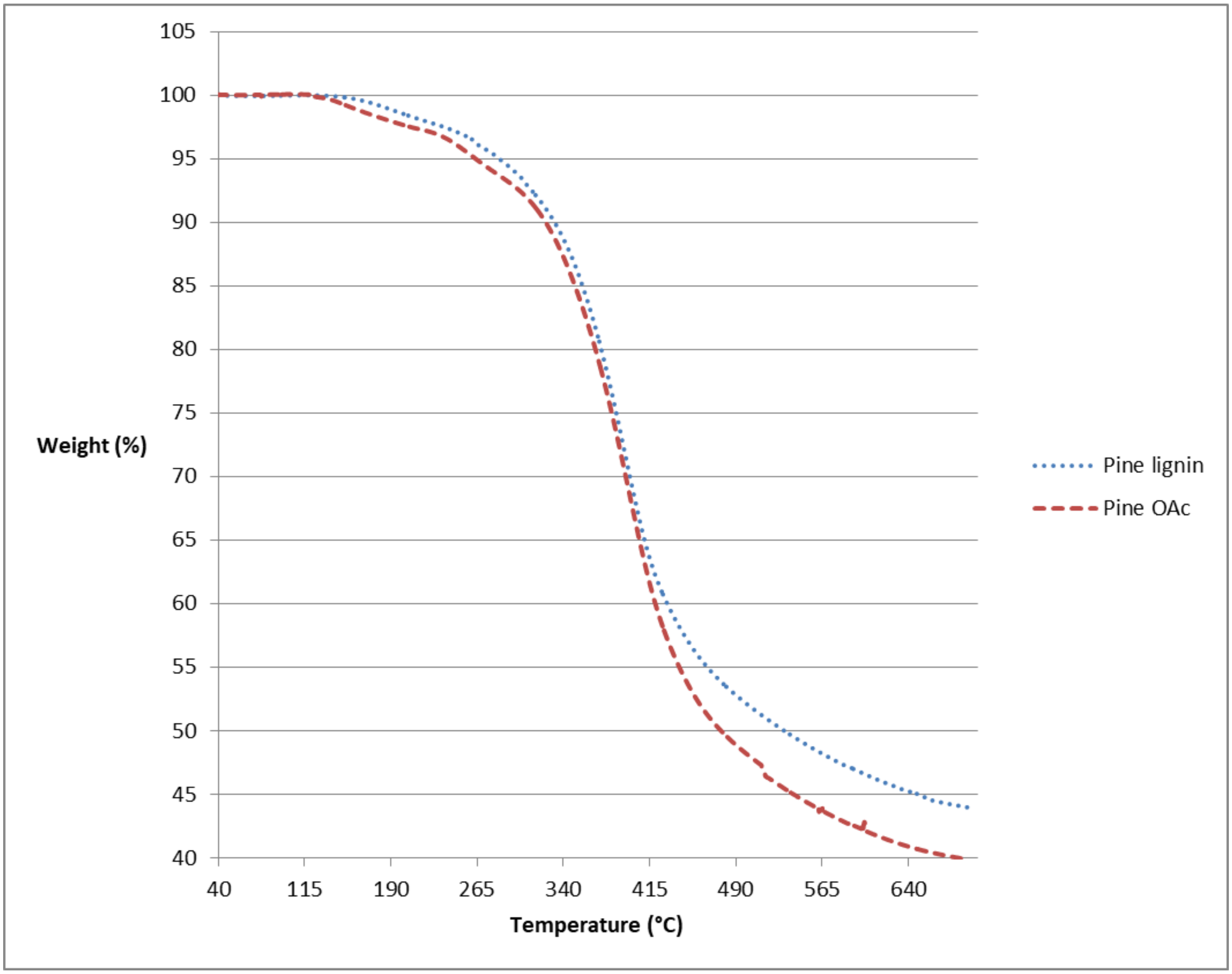

Figure S16. TGA of pine lignin and acetylated pine lignin after extensive drying
